# New Invertebrate Vectors for PST, Spirolides and Okadaic Acid in the North Atlantic

**DOI:** 10.3390/md11061936

**Published:** 2013-06-05

**Authors:** Marisa Silva, Aldo Barreiro, Paula Rodriguez, Paz Otero, Joana Azevedo, Amparo Alfonso, Luis M. Botana, Vitor Vasconcelos

**Affiliations:** 1 Department of Biology, Faculty of Sciences, University of Porto, Rua do Campo Alegre, Porto 4619-007, Portugal; E-Mails: marisasilva17@gmail.com (M.S.); aldo.barreiro@gmail.com (A.B.); 2 Center of Marine and Environmental Research—CIMAR/CIIMAR, University of Porto, Rua dos Bragas 289, Porto 4050-123, Portugal; E-Mail: joana_passo@hotmail.com; 3 Department of Pharmacology, Faculty of Veterinary, University of Santiago of Compostela, Lugo 27002, Spain; E-Mails: paula.rodriguez17@rai.usc.es (P.R.); mariapaz.otero@rai.usc.es (P.O.); amparo.alfonso@usc.es (A.A.); Luis.Botana@usc.es (L.M.B.); 4 Department of Chemical and Biomolecular Sciences, School of Health and Technology of Porto, Vila Nova de Gaia 4400-330, Portugal

**Keywords:** new vectors, PST, okadaic acid, spirolides, North Atlantic

## Abstract

The prevalence of poisoning events due to harmful algal blooms (HABs) has declined during the last two decades through monitoring programs and legislation, implemented mainly for bivalves. However, new toxin vectors and emergent toxins pose a challenge to public health. Several locations on the Portuguese coast were surveyed between 2009 and 2010 for three distinct biotoxin groups [saxitoxin (PST), spirolide (SPX) and okadaic acid (OA)], in 14 benthic species of mollusks and echinoderms. Our main goals were to detect new vectors and unravel the seasonal and geographical patterns of these toxins. PSTs were analyzed by the Lawrence method, SPXs by LC-MS/MS, and OA by LC-MS/MS and UPLC-MS/MS. We report 16 new vectors for these toxins in the North Atlantic. There were differences in toxin contents among species, but no significant geographical or seasonal patterns were found. Our results suggest that legislation should be adjusted to extend the monitoring of marine toxins to a wider range of species besides edible bivalves.

## 1. Introduction

The occurrence of harmful algal blooms (HABs) has been increasing globally throughout the 20th century [1]. Rising water temperature and eutrophication were pointed out as the most significant factors in this increase [2]. HABs have a severe impact on the economy and human health, because phycotoxins may travel along the food chain, contaminating edible shellfish, resulting in intoxication incidents [3,4,5,6,7]. These phycotoxins can be classified as being either hydrophilic or lipophilic, with typical molecular weights (MW) of 500 Da and 600 Da, respectively [8]. Besides commercially important bivalves, other organisms, such as crustaceans, gastropods and fish, have been reported as phycotoxin vectors [9,10,11]. HAB poisoning events have been sporadic worldwide during the past two decades. The establishment of legislation, mostly for edible bivalves and monitoring programs are of extreme importance for poisoning prevention [7,12,13].

Paralytic shellfish toxins (PSTs) are a group of alkaloids that cause Paralytic Shellfish Poisoning (PSP). Intoxication events caused by these toxins result from the ingestion of contaminated shellfish [4,5]. These alkaloids are comprised of saxitoxin (STX) plus 57 analogs that bind specifically to site 1 of voltage-gated Na^+^ channels (Nav), causing paralysis. Currently, there is no antidote available. Ventilation support plus fluid therapy are the only treatments available [14]. After the first report in the USA in 1920, PSTs have been reported all over the globe [4,5]. The main producers of these neurotoxins are several species of dinoflagellates (*Alexandrium* sp., *Gymnodinium catenatum* and *Pyrodinium bahamense* var. *compressum*), as well as certain brackish and freshwater cyanobacteria (*Anabaena circinalis*, *A.* *lemmermannii*, *Aphanizomenon gracile*, *A. issatschenkoi*, *Rivularia* sp., *Lynbya wolleii*, *Planktothrix* sp. and *Cylindrospermopsis raciborskii*) [15,16,17,18,19]. The European Food Safety Authority (EFSA), proposes the use of toxicity equivalency factors (TEFs) as a unit that integrates all PST analogues for their conversion to SXT equivalents [14]. Fourteen TEF values have been calculated based on acute intra peritoneal (i.p.) toxicity in mice: NEO = 1; GTX_1_ = 1; dc-STX = 1; GTX_4_ = 0.7; GTX_3_ = 0.6; GTX_2_ = 0.4; dc-NeoSTX = 0.4; dc-GTX_3_ = 0.4; 11-hydroxy-STX = 0.3; dc-GTX_2_ = 0.2; GTX_5_ = 0.1; GTX_6_ = 0.1; C_2_ = 0.1; C_4_ = 0.1 [14]. There are only 15 standards available as certified material. In 2009, EFSA established the acute reference dose (ARfD) for PSTs as 0.5 μg STX equivalents/kg body weight, due to the lack of repeated oral intake data of these toxins in humans and animals [14]. The guideline limit value for PSTs is 800 ng SXT equivalents/g shellfish meat [20].

Spirolides (SPXs) belong to the group of the cyclic imines that includes other compounds, like gymnodimines, pinnatoxins, pteriatoxins, symbioimines, prorocentrolides and spiro-prorocentrolides [21,22]. These marine toxins have a cyclic imine group that is responsible for their neurotoxicity. SPXs bind specifically to both the muscarinic and nicotinic acetylcholine receptors (mAChR and nAChR, respectively) in the central and peripheral nervous system [23]. These biotoxins have acute toxicity by i.p. and oral administration in mouse bioassays (MBAs); however, there have been no reports of poisoning incidents [23,24]. SPXs have already been reported in microalgae and shellfish in Canada [25,26,27], Scotland [28], USA [29], Norway [30], Italy [31], Denmark [21], France [32], Spain [33] and Chile [34]. Other cyclic imines seem to be confined to warmer waters of the Pacific Ocean, with reports from Japan, China and New Zealand [23,35]. SPXs are produced by dinoflagellate species, such as *Alexandrium ostenfeldii* and *A. peruvianum* [36]. The C group is the most toxic among SPXs and the only one with certified material available [37]. There are no legislated limits for cyclic imines, due to the lack of toxicological data [38].

Okadaic acid (OA) and its derivatives, dinophysistoxin-1 and 2 (DTX-1, DTX-2), are responsible for Diarrheic Shellfish Poisoning (DSP). This group of neurotoxins is composed of polyethers that inhibit type 1 and 2A serine/threonine phosphatases [39]. OA and its analog, DTX-1, are also tumor promoters [40]. DSP incidents have been reported all over the globe [41,42,43,44]. Several species of phytoplankton have already been reported as causing DSP, particularly from the genera *Phalacroma*, *Prorocentrum* and *Dinophysis* [45,46,47,48,49]. The accepted limit established in Europe is 160 ng OA equivalents/g shellfish meat [20].

The occurrence of emergent toxins and new vectors in the Atlantic Ocean brings new challenges for monitoring programs. It is necessary to fill the lack of knowledge and improve monitoring programs in order to minimize risks to human health. Close monitoring, in conjunction with reliable detection methods, helps to reduce the number of poisonings. Benthic organisms are generally poorly studied regarding their role as potential vectors for marine toxins, with the exception of bivalves [3,4,5,7,13,26,37]. Several benthic species were surveyed in this study, including gastropods (sea-snails, sea-slugs and limpets), bivalves (mussels) and echinoderms (starfishes and sea-urchins). We have chosen these particular species, because most of them are edible and we strongly believe that the human health risk is underestimated. In addition, all of them play an important role in benthic food-chains. Our aims were to unravel new vectors of PST, OA and SPX, to study the existence of seasonal, geographical and interspecific patterns of toxin accumulation. Table 1 shows the species sampled in this work and their trophic level, edibility and monitoring status.

**Table 1:** Species sampled and their trophic level, edibility and monitoring status.

Species	Trophic level	Edibility	Monitored	References
*Gibbula* sp.	Grazer	Yes	No	[20,50]
*Monodonta* sp.	Grazer	Yes	No	[20,50]
*Littorina* sp.	Grazer	Yes	No	[20,50]
*Patella intermedia*	Grazer	Yes	No	[20,51]
*Paracentrotus lividus*	Grazer	Yes	No	[20,52]
*Echinus esculentus*	Grazer	Yes	No	[20,53]
*Aplysia depilans*	Grazer	No	No	[20,54]
*Mytilus galloprovincialis*	Filter feeder	Yes	Yes	[20,51]
*Nucella lapillus*	1st level Predator	Yes	No	[20,51]
*Marthasterias glacialis*	2nd level Predator	No	No	[20,51]
*Charonia lampas*	3rd level Predator	Yes	No	[20,55]

## 2. Results and Discussion

### 2.1. PSTs

**Figure 1:**
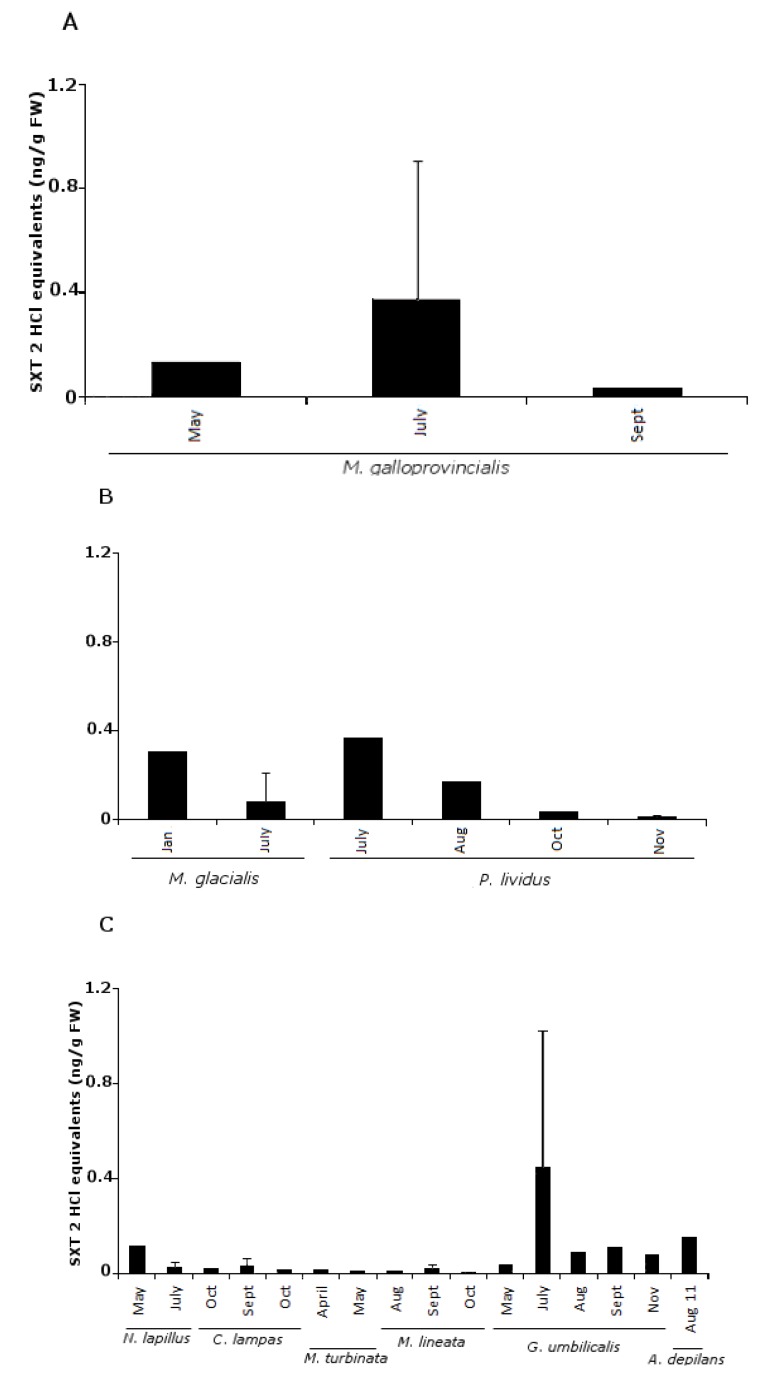
Paralytic shellfish toxin (PST) content in saxitoxin (STX) 2 HCl equivalent ng/g fresh weight (fw) in all groups: (**A**) bivalves—*Mytilus galloprovincialis*; (**B**) echinoderms—*Marthasterias glacialis* and *Paracentrotus lividus*; (**C**) gastropods—*Nucella lapillus*; *Charonia lampas*; *Monodonta turbinata*; *Monodonta lineata*; *Gibbula umbilicalis* and *Aplysia depilans*. Limit value for PSTs is 800 ng SXT equivalents/g shellfish meat.

**Figure 2:**
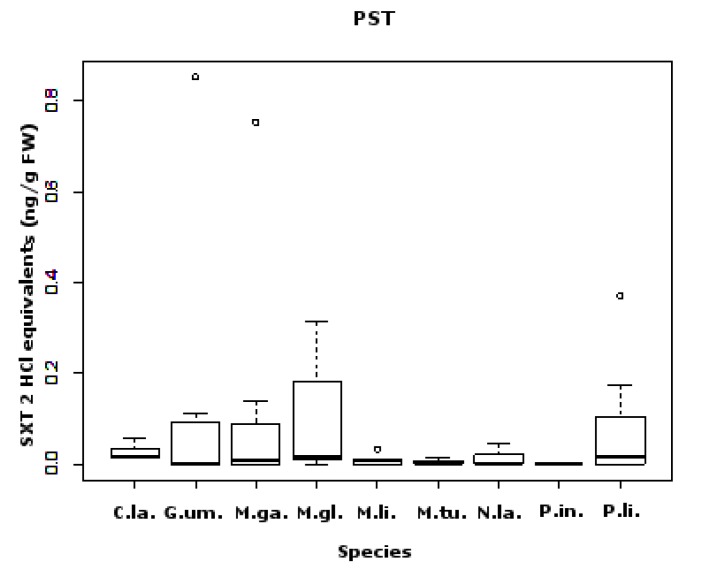
Box and whisker plots for PST concentrations [SXT HCl 2 equivalents (ng/g fw)] found in each species (*C.* *la.*—*C. lampas*; *G.* *um.*—*G. umbilicalis*; *M.* *ga.*—*M. galloprovincialis*; *M.* *gl.*—*M. glacialis*; *M.* *li.*—*M. lineata*; *M.* *tu.*—*M. turbinata*; *N.* *la.*—*N. lapillus*; *P.* *in.*—*P. intermedia*; *P.* *li.*—*P. lividus*). Limit value for PSTs is 800 ng SXT equivalents/g shellfish meat.

**Figure 3:**
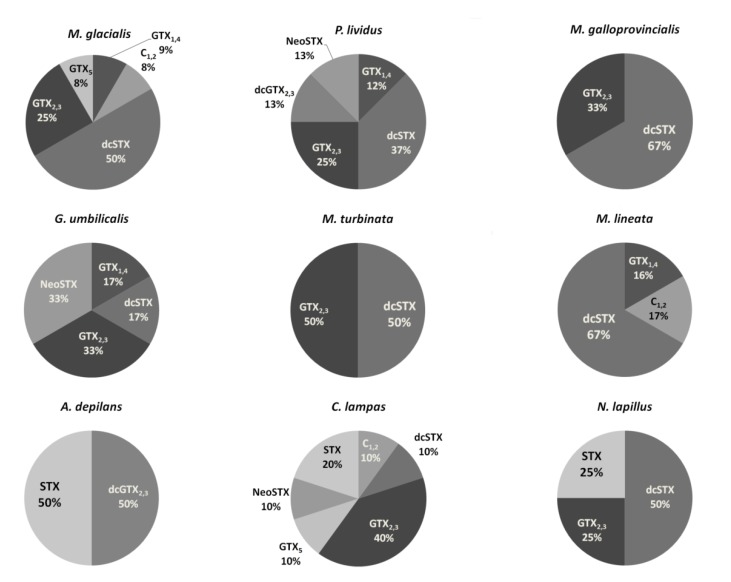
PST profiles in each group screened.

In 104 samples analyzed, we obtained 51% positive results. A sample was considered positive when the toxin levels detected were above the limit of detection (LOD); however, 30.2% of these samples were below the limit of quantification (LOQ) for SXT and its analogs (Figure 1). Regarding gastropods, bivalves and echinoderms, most of the positive results occurred in the late spring-summer season. In the general linear model, significant differences were not detected for any of the fixed factors: sampling site (*F*_11,90_ = 1.9; *p* = 0.08) or species (*F*_9,92_ = 1.8; *p* = 0.09). These results can be explained by the high resemblance between species average values, although there were differences in their dispersion. It should be noted that the average PST concentration was in general very low (Figure 2). The percentage of analogs in each group screened (Figure 3) shows that the most toxic groups, carbamate (STX, NEO, GTX1, GTX2, GTX3 and GTX4) and decarbamoyl (dcSTX, dcGTX1, dcGTX2, dcGTX3 and dcGTX4), were always present in all species [14]. The former group was the most common, since 67% of the positives have carbamate forms, constituting 50%–83% of the PSTs’ total content. The decarbamoyl group contributes from 50% to 67% of the total toxin content in the majority of the positive samples. The less toxic *N*-sulfo-carbamoyl group (C1, C2, C3, C4, GTX5 and GTX6) was only detected in 33% of the positive samples, reaching a maximum of 20% of the total PST content.

In all the species, positives were below the limit implemented in Europe [20]. In comparison with recent work performed on the Portuguese coast [4], the present toxin profile detected was wider, showing a higher prevalence of the most potent analogs. However, the detected concentrations were lower. There was no previous data available regarding PSTs on the Portuguese coast in taxa other than bivalves [13,56]. PST metabolism in bivalves is very complex, since some species show the ability to convert low toxic forms to high toxic ones, therefore presenting different toxin profile than the producers [57,58,59]. Gastropods are known for their ability to accumulate PSTs and having low depuration rates and, thus, being quite dangerous to humans, since they remain toxic for long periods of time. Most poisoning events due to gastropod ingestion have been reported in Eastern Asia [9,11]. Regarding bivalves, *M. galloprovincialis* has 67% of its PSTs as dcSTX, with the remainder being GTX_2,3_ (Figure 3). This profile could be due either to a high carbamoylase activity or feeding on more toxic dinoflagellate species [9], in contrast to a previous work [13], which showed mussels with a toxin profile enriched in the less toxic groups. There is evidence that GTX analogs can be converted into SXT in shellfish, due to the action of bacteria [60], and mediated by glutathione (GSH) [61]. This could be the reason why the mussel predator, *N. lapillus*, has 25% of SXT. Nevertheless, grazer gastropods can also acquire PSTs directly [62]. This is the case with *Monodonta* sp., *G. umbilicalis* and *A. depilans*, with the latter showing a higher toxin content (50% STX). *Gibbula umbilicalis* had 30% of NEO, and in *Monodonta* species, the carbamate group was absent (Figure 3). *Charonia lampas* is a scavenger and a predator, so it most probably acquired PSTs indirectly. In comparison with other works, the levels found in this species were low [63]. Although PSTs have already been detected in echinoderms, namely in starfish [64,65], the report of PSTs in sea-urchins is a novelty. Comparing the toxin content of both species, *M. glacialis* had 84% of its toxin content as carbamate analogs, while *P. lividus* had 50%, which could be due to a dietary effect. Asakawa *et al.* (1997) [64] found higher concentrations of PSTs in the starfish *Asterias amurensis*; however, in this work, we detected a higher diversity and concentration of the carbamate group.

Our data show six new PST vectors among the species screened: four gastropods (*G. umbilicalis*, *N. lapillus*, *Monodonta* sp. and *A. depilans*) and two echinoderms (*P. lividus*, *M. glacialis*).

### 2.2. OA

**Figure 4:**
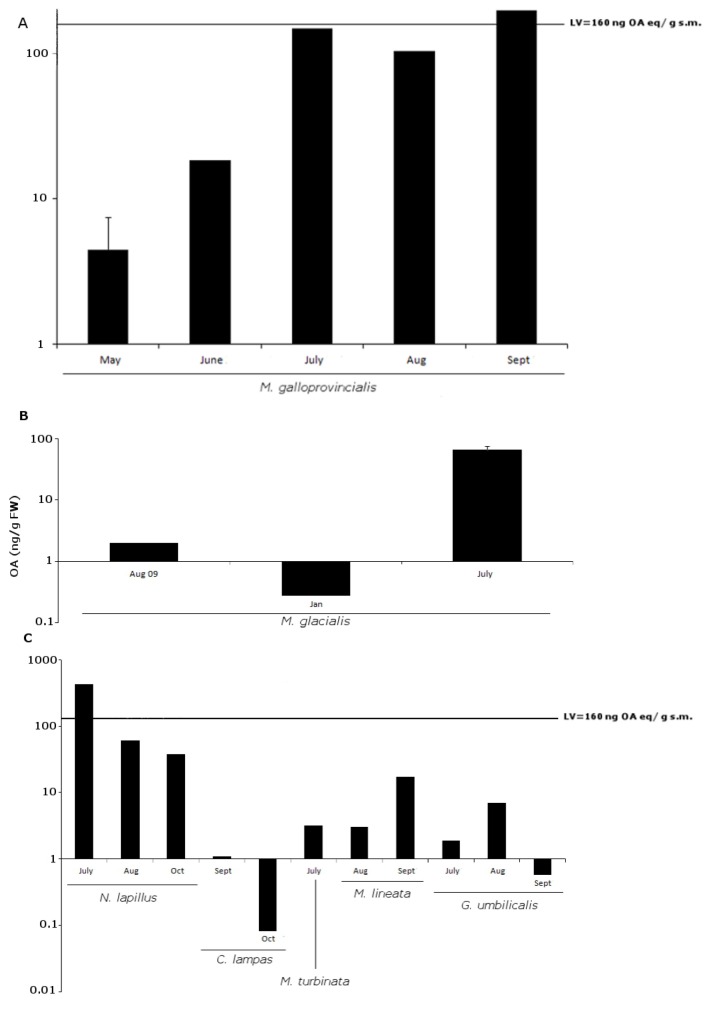
Okadaic acid (OA) positive results (ng/g fw) for all sampled groups of organisms: (**A**) bivalves; (**B**) echinoderms; (**C**) gastropods. Y-axis in logarithmic scale. Limit value (LV) established in Europe is 160 ng OA equivalents (eq)/g shellfish meat (s.m.).

A total of 51% of 55 samples were positive for OA (>LOD), with 14.3% of these below LOQ. No analogs were detected. Most of the positives (61.5%), as well as the higher concentrations detected in the late spring-summer season (Figure 4). The average concentration found for each species ranged between 0.58 ng/g fw (in *C. lampas*) and 175.71 ng/g fw (*N. lapillus*). Statistically significant differences for sampling site were detected, although they were close to the limit of statistical significance (*F*_8,43_ = 2.4; *p* = 0.049). This was possibly due to the fact that OA had a greater proportion of positive results and higher toxin concentration. It is possible that a greater sampling effort for PSTs and SPX could result in significant *p*-values for the sampling site as well. For species, clear significant differences were found (*F*_8,43_ = 2.4; *p* < 0.001).

**Figure 5:**
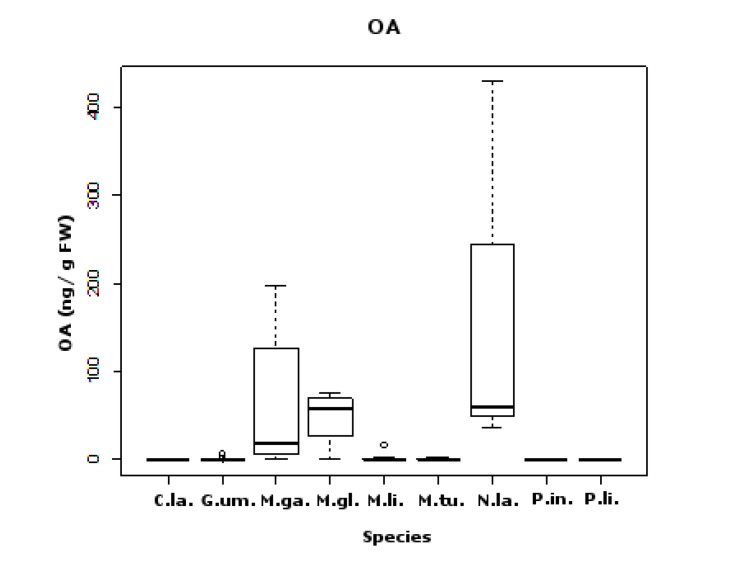
Box and whisker plots of OA concentrations (ng/g fw) found in each species (*C.* *la.*—*C. lampas*; *G.* *um.*—*G. umbilicalis*; *M.* *ga.*—*M. galloprovincialis*; *M.* *gl.*—*M. glacialis*; *M.* *li.*—*M. lineata*; *M.* *tu.*—*M. turbinata*; *N.* *la.*—*N. lapillus*; *P.* *in.*—*P. intermedia*; *P.* *li.*—*P. lividus*). Limit value (LV) established in Europe is 160 ng OA equivalents (eq)/g shellfish meat (s.m.).

**Figure 6:**
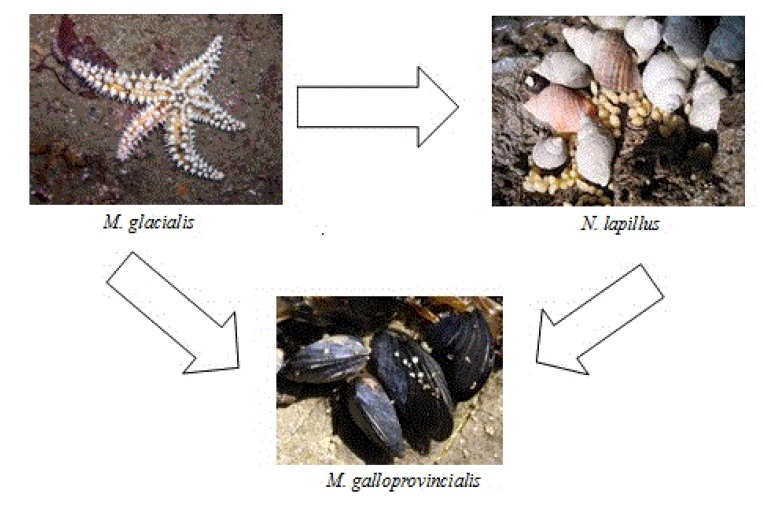
Trophic relation between *M. galloprovincialis*, *N. lapillus* and *M. glacialis*.

The majority of significant differences in pairwise comparisons between species appeared in this group of toxins. Differences were due to two major groups; those that have the lowest average OA values (six species—*C. lampas*, *G. umbilicalis*, *M. lineata*, *M. turbinata*, *P. lividus*, *P. intermedia*) *versus* those that have the highest average values (three species—*M. glacialis*, *M. galloprovincialis*, *N. lapillus*) (see Figure 5 and Table 2). Interestingly, the species with highest average OA values are linked through the food chain, with *M. galloprovincialis* being predated by *N. lapillus* and both by *M. glacialis* (Figure 6) [51]. We show five first reports of OA in *G. umbilicalis*, *N. lapillus*, *Monodonta* sp., *P. lividus* and *M. glacialis*. These results suggest the need for a revision of marine toxin monitoring policies in order to consider the inclusion of groups other than bivalves. In addition, the highest concentration detected did not occur in bivalves, but in the edible gastropod, *N. lapillus*.

**Table 2:** Species’ OA concentration pairwise comparisons using *post hoc* Tukey test.

Species pairwise comparisons	*z*	*p*
*N.* * lapillus–C.* * lampas*	3.3	0.03
*M.* * glacialis–G.* * umbilicalis*	3.8	<0.01
*M.* * galloprovincialis–G.* * umbilicalis*	5.2	<0.001
*N.* * lapillus–G.* * umbilicalis*	4.7	<0.001
*M.* * lineata–M.* * glacialis*	−3.6	<0.01
*M.* * turbinata–M.* * glacialis*	−3.4	0.02
*P.* * lividus–M.* * glacialis*	−5.6	<0.001
*P.* * intermedia–M.* * glacialis*	−5.5	<0.001
*M.* * galloprovincialis–M.* * lineata*	4.8	<0.001
*N.* * lapillus–M.* * lineata*	4.6	<0.001
*M.* * galloprovincialis–M.* * turbinata*	4.1	<0.01
*N.* * lapillus–M.* * turbinata*	4.0	<0.01
*P.* * lividus–M.* * galloprovincialis*	−7.1	<0.001
*P.* * intermedia–M.* * galloprovincialis*	−6.4	<0.001
*P.* * lividus–N.* * lapillus*	−6.4	<0.001
*P.* * intermedia–N.* * lapillus*	−5.9	<0.001
*P.* * intermedia–P.* * lividus*	−0.6	<0.001

Two samples exceeded the 160 ng OA equivalents/g limit [20]: *N. lapillus* collected in July 2010 in Memória with 429.41 ng/g fw and *M. galloprovincialis* collected in September 2010 from the same site with 198.17 ng/g fw. Our average OA levels detected were similar to other reports on the Portuguese coast [13]. We detected OA in January, which seemed to be unusual [13], but this could be explained by our wider range of species screened.

### 2.3. 13-Desmethyl Spirolide C

**Figure 7:**
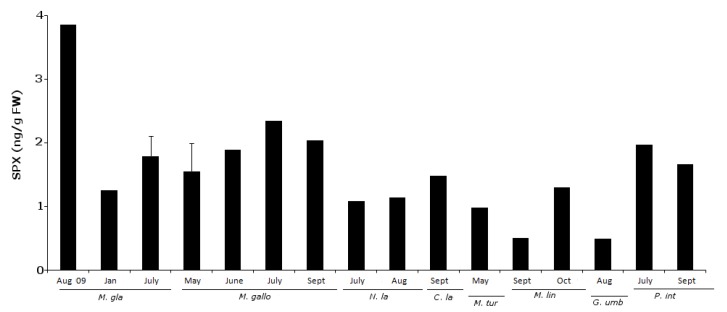
SPX positive results (ng/g fw) for all sampled groups of organisms.

In 55 samples analyzed, 38.2% of the results were positive for 13-desmethyl Spirolide C (Figure 7). A sample was considered positive when the toxin levels detected were above the LOD. Concentrations ranged between 0.49 ng/g fw and 3.86 ng/g fw. Approximately half (48%) of the positive results occurred in the late spring-summer season. Concentrations were also higher in this period (Figure 7).

**Figure 8:**
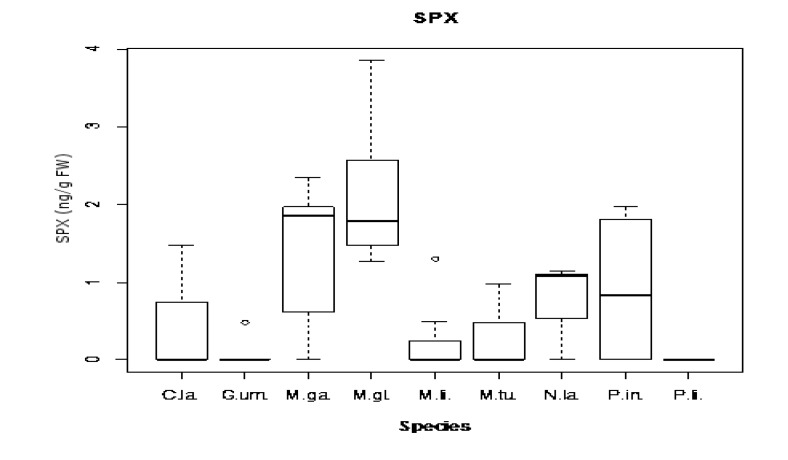
Box and whisker plots of SPX concentration (ng/g fw) found in each species (*C.* *la.*—*C. lampas*; *G.* *um.*—*G. umbilicalis*; *M.* *ga.*—*M. galloprovincialis*; *M.* *gl.*—*M. glacialis*; *M.* *li.*—*M. lineata*; *M.* *tu.*—*M. turbinata*; *N.* *la.*—*N. lapillus*; *P.* *in.*—*P. intermedia*; *P.* *li.*—*P. lividus*).

Regarding SPX toxin concentrations, significant differences were found for between species, but not for sampling sites. Here, as for OA, differences in pairwise comparisons were due to the same differentiated groups, with the exception of the species *C. lampas* and *P. intermedia*, as shown in Figure 8. Both species showed a large dispersion (see Table 3). We have shown five first reports for this biotoxin in *G. umbilicalis*, *N. lapillus*, *Monodonta* sp., *M. glacialis* and *P. intermedia*. It is important to point out that this is not only the first report of SPX in these species, but also on the Portuguese coast. SPX showed a wide range of dispersion, not only geographical, but also in terms of vectors.

**Table 3:** Species’ SPX concentration pairwise comparisons using *post hoc* Tukey test.

Species pairwise comparisons	*z*	*p*
*M.* * glacialis–G.* * umbilicalis*	4.3	<0.001
*M.* * galloprovincialis–G.* * umbilicalis*	3.2	0.02
*M.* * lineata–M.* * glacialis*	−3.9	<0.01
*M.* * turbinata–M.* * glacialis*	−3.5	0.01
*P.* * lividus–M.* * glacialis*	−4.2	<0.001
*P.* * lividus–M.* * galloprovincialis*	−3.1	0.04

### 2.4. Toxins Concentrations Found: Comparison between Species and Sampling Sites

The results of the MANOVA test show that there were clear significant differences between species (*F*_8,42_ = 2.4; *p* < 0.001) and small differences between sampling sites (*F*_8,42_ = 1.7; *p* = 0.04). These were, in general, the same results as those from the analysis for each individual toxin. In the pairwise multivariate comparisons performed with the Hotelling test, generally, the species that showed more significant differences regarding toxin concentrations were *M. glacialis*, *M. galloprovincialis* and *N. lapillus*. The previous Tukey tests showed a similar pattern, *i.e.*, the same pairs of species showed the most significant differences (see Table 4). With respect to sampling site, differences between sites were, as expected, from the test for individual toxins, mainly due to OA (Table 4). However, if the sampling effort was more balanced, differences between sampling sites could also be detected for PSTs and SPX. Memória was the most intensively sampled site and showed more differences in this test. It should be noted that the species with the highest toxin concentrations are linked by the food-chain. *Mytilus galloprovincialis* is predated by both *N. lapillus* and *M. glacialis*, while the latter also preys on *N. lapillus* [51,66]. The advantage of this multivariate test is that our data support the possibility of bioaccumulation of toxins along the food chain, taking into account all toxins together.

**Table 4:** Pairwise comparisons between factor levels of sampling site and species, using the three groups of toxins as dependent factors.

Pairwise Comparison	*p*
*C.* * lampas–P.* * intermedia*	0.01
*G.* * umbilicalis–M.* * glacialis*	<0.001
*G.* * umbilicalis–M.* * galloprovincialis*	0.03
*G.* * umbilicalis–N.* * lapillus*	<0.01
*P.* * intermedia–M.* * glacialis*	<0.001
*P.* * intermedia–M.* * galloprovincialis*	0.02
*P.* * intermedia–N.* * lapillus*	<0.001
*M.* * lineata–M.* * glacialis*	0.01
*M.* * turbinata–M.* * glacialis*	<0.01
*M.* * galloprovincialis–P.* * lividus*	<0.001
*P.* * lividus–M.* * glacialis*	0
*P.* * lividus–N.* * lapillus*	
Angeiras–Memória	0.03
Memória–Porto Côvo	0.01
Memória–Viana do Castelo	0.03
Memória–Almograve	0.01

## 3. Experimental Section

### 3.1. Selected Species and Sampling Points

In this study, we were searching for unconventional vectors of marine toxins, so we surveyed several benthic species. We focused our sampling on edible species, but also other species that play an important role in the food-chain: gastropods (*Monodonta lineata*, *Monodonta turbinata*, *Gibbula umbilicalis*, *Gibbula magus*, *Littorina littorea*, *Littorina saxatilis*, *Nucella lapillus*, *Patella intermedia*, *Aplysia depilans*, *Charonia lampas*), bivalves (*Mytilus galloprovincialis*), sea-urchins (*Paracentrotus lividus*, *Echinus esculentus*) and starfish (*Marthasterias glacialis*).

**Figure 9:**
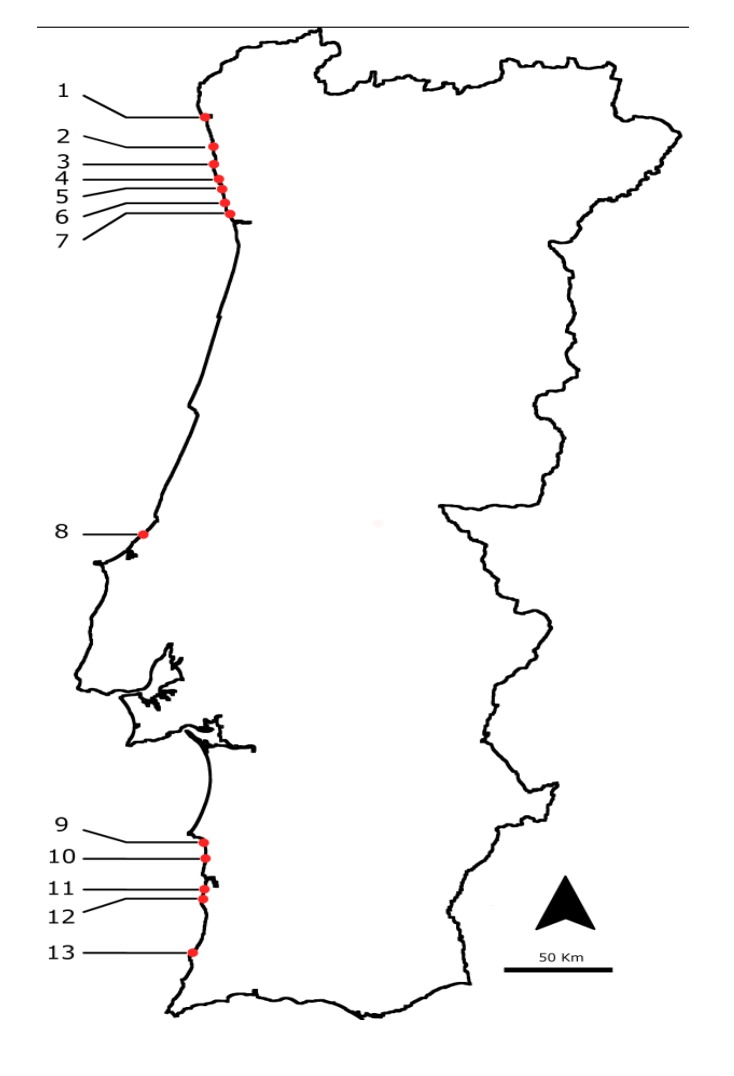
Location of the sampling points on the Atlantic Portuguese coast: 1: Viana do Castelo; 2: Esposende; 3: Póvoa do Varzim; 4: Angeiras; 5: Memória; 6: Valadares; 7: Aguda; 8: São Martinho do Porto; 9: São Torpes; 10: Porto Côvo; 11: Monte Clérigos; 12: Vila Nova de Milfontes; 13: Almograve.

Animals were collected monthly from several locations along the northern and southern Portuguese coast (Figure 9) from July 2009 till the end of 2010. Samples of *Charonia lampas* were purchased in local markets from the same areas. Organisms were collected from the intertidal area during low tides and were transported to the laboratory in refrigerated containers. If they were not processed immediately, they were frozen at −20 °C. The number of samples collected and average number of specimens needed to set a pooled sample are detailed in Table 5.

**Table 5:** Average number of specimens comprising a pooled sample and number of samples collected since July 2009 till 2010. Availability of animals is dependent on their geographical distribution and ecology.

Species	Number of pooled samples collected from July 2009 till end 2010	Average number of animals collected to set a pooled sample
*Gibbula umbilicalis*	34	100
*Gibbula magus*	1	90
*Monodonta lineata*	20	86
*Monodonta turbinata*	21	86
*Nucella lapillus*	13	15
*Littorina littorea*	2	10
*Littorina saxatilis*	4	15
*Patella intermedia*	4	15
*Charonia lampas*	5	1
*Mytilus galloprovincialis*	7	30
*Paracentrotus lividus*	10	10
*Marthasterias glacialis*	8	1
*Echinus esculentus*	2	1
*Aplysia depilans*	1	1

### 3.2. Sample Preparation

#### 3.2.1. Paralytic Shellfish Toxin (PST) Extraction

Samples were extracted following the Lawrence *et al.* method [67]. Animals were dissected and homogenized with a blender (A320R1, 700 W, Moulinex) in pooled groups in order to obtain 1 g of extractable tissue, with the exception of *Charonia lampas*, *Marthasterias glacialis*, *Echinus esculentus* and *Aplysia depilans*, which were treated individually. The homogenized tissue was extracted with 3 mL 1% acetic acid (Merck, Portugal) using a vortex mixer for 5 min (Top Mix 1118, Fisher Scientific, Portugal), then heated in a boiling water bath for 10 min, remixed on a vortex mixer and placed in ice for 5 min. The resulting mixture was sonicated (1 min, 70 Hz, Vibra Cell, Sonic & Materials, USA) and subsequently centrifuged at 4495× *g* for 10 min at 4 °C (Centrifugal-Legend RT, Sorvall). Then, 2 mL of 1% acetic acid was added to the centrifuge tube containing the solid residue, mixed well on a vortex mixer and centrifuged again (4495 *g*/10 min/4 °C). The supernatant solution was collected pooled into the same tube that contained the first portion of extract and finally diluted to 5 mL with 1% acetic acid. An aliquot (1 mL) of the crude extract was passed through a C18 solid-phase extraction (SPE) cartridge (500 mg/3 mL volume from Supelco, Bellefonte, PA, USA), previously conditioned with 10 mL methanol (Fisher Scientific, Leics, UK), followed by 10 mL water (MilliQ). Toxin was absorbed onto the column and eluted at a flux of 0.4 mL/min using 2 mL of ultrapure water. The pH was adjusted to 6.5 using 0.2 M NaOH (Sigma Aldrich, Portugal). The eluents were evaporated to dryness (Acid-resistant Centrivap Concentrator, Labconco, MO, USA) and dissolved in 0.5 mL of 0.03 M acetic acid. The extracts were passed through 0.45 μm filters (Ultrafree-MC centrifugal filter devices from Millipore, Spain) and pre-oxidized before HPCL-FLD analysis. The extracts were oxidized with hydrogen peroxide and periodate (Panreac Quimica, Spain). Peroxide oxidation consisted of mixing 25 μL of 10% hydrogen peroxide in water (v/v) with 250 μL 1 M NaOH and 100 μL of sample. The solution was then mixed on a vortex mixer and rested at room temperature for 2 min. Then 20 μL of acetic acid was added. At this point, the solution was homogenized and 25 μL was injected into the HPLC system. Peroxide oxidant solution was prepared daily. Periodate oxidation consisted of mixing 100 μL sample with 100 μL of deionized water and 500 μL periodate oxidant. The solution reacted for 1 min at room temperature before the addition of 5 μL of acetic acid. Periodate oxidant was prepared daily by mixing 5 mL of 0.03 M periodic acid with 5 mL of 0.3 M ammonium formate and 5 mL of 0.3 M sodium phosphate dibasic. The pH of the final solution was adjusted to 8.2 with 1 M NaOH.

#### 3.2.2. Lipophilic Toxins Extraction

The protocol from Otero *et al.* (2010) [68] was followed for the extraction of okadaic acid (OA), dinophysistoxin (DTX-1 and DTX-2) and spirolides (13,19-didesmethyl SPX C; 13-desmethyl SPX C). Animals were dissected and homogenized with the help of a blender (A320R1, 700 W, Moulinex) in pooled groups in order to obtain 1 g of extractable tissue, with the exception of *Charonia lampas*, *Marthasterias glacialis*, *Echinus esculentus* and *Aplysia depilans*. In these cases, each animal was treated separately. The 1 g of homogenized tissue was extracted with 3 mL of methanol (Fisher Scientific), then centrifuged during 10 min at 2932 *g* at 4 °C (Centrifugal-Legend RT). This procedure was repeated twice, and the supernatants combined and concentrated to dryness (Acid-resistant Centrivap Concentrator, Labconco). Residues were then re-suspended in 10 mL of water (MilliQ) and partitioned twice against dichloromethane (Merck). The organic layers (20 mL) were reserved and concentrated by drying and re-suspended in 1 mL of methanol. Then 500 μL was concentrated to dryness, re-suspended in 100 μL of methanol and filtered through a 0.45 μm filter (UltraFree-MC centrifugal devices, Millipore) before LC-MS/MS analysis.

### 3.3. Sample Analysis

#### 3.3.1. PSP HPLC-FLD Conditions

**Figure 10:**
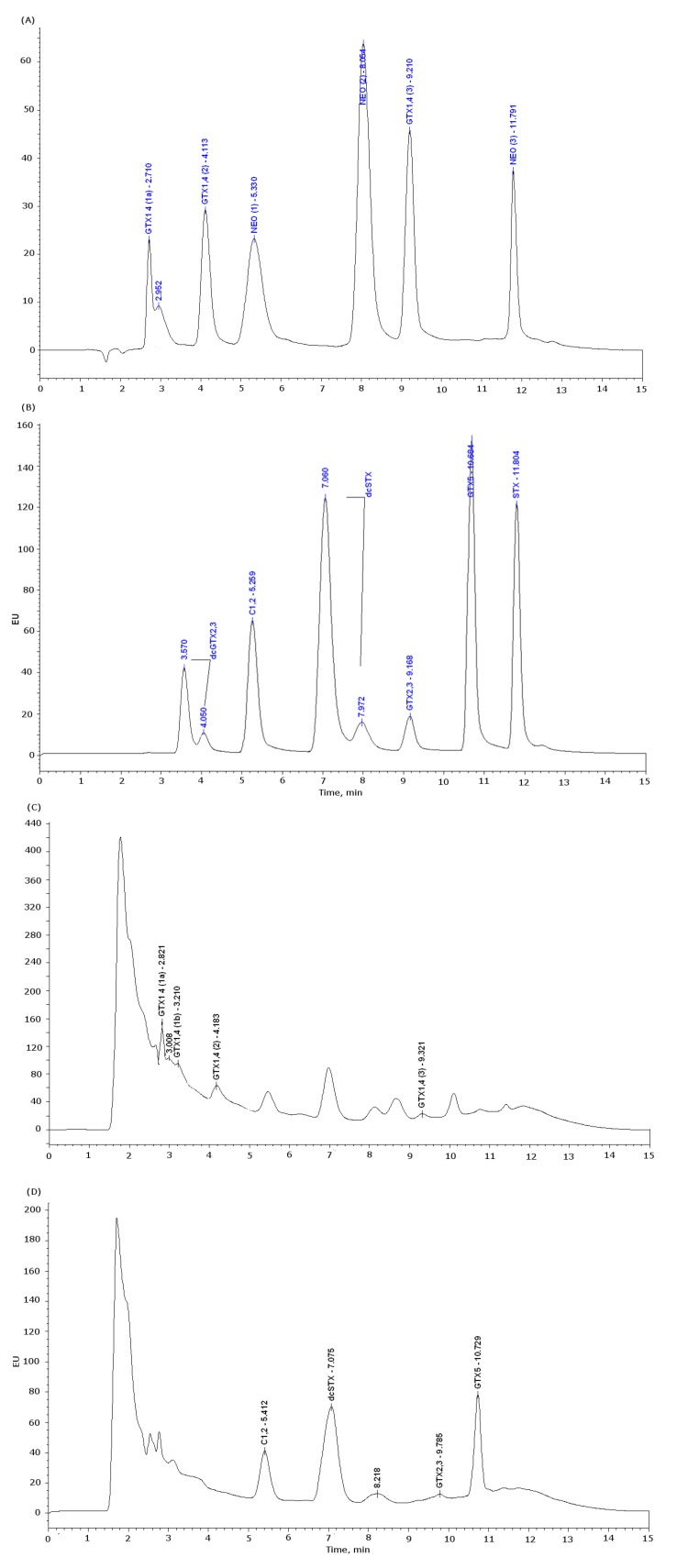
Chromatograms of the PSTs standards and a positive sample for *M. glacialis*, hydroxylated toxins are oxidized with periodate and non-hydroxylated toxins with peroxide. (**A**) Standards injected with periodate oxidation, peak GTX_1,4_(1) and GTX_1,4_(3) are secondary oxidation products of GTX_1,4_. Peaks NEO(1) and NEO(3) are secondary oxidation products of NEO. (**B**) Standards injected with peroxide oxidation. (**C**) Chromatogram of a positive sample in *M. glacialis* injected with periodate oxidation. (**D**) Chromatogram of a positive sample in *M. glacialis* injected with peroxide oxidation.

The conditions were the same as reported by Rodriguez *et al.* (2010) [69]. Briefly, the analyses were performed using high-performance liquid chromatography with fluorescence detection (HPLC-FLD) equipment (Waters 2695), consisting of a pump (Waters 515) and a column (SupelcosilTM LC-18, 5 μm, 15 × 4.6 mm, Sigma Aldrich) kept at 35 °C. Empower software (Waters, Manchester, UK) was used to control the process. Toxins were detected with a Waters 2475 fluorescence detector, with excitation set to 340 nm and emission to 395 nm. Injection volume was 25 μL. Eluent (A) of the mobile phase was composed by 0.1 M of ammonium formate (Sigma Aldrich). Eluent (B) was composed by 0.1 M of ammonium formate in 5% of acetonitrile (Panreac Quimica). Both eluents had their pH adjusted to 6 with 1 M acetic acid. The gradient started with 5% of mobile phase (B) for the first 5 min, then increasing to 70% up to min 9, then decreasing to 0% in min 11 and stayed in 0% till the end of the run (15 min). The flow rate was 1 mL/min. For toxin detection and determination, standards of SXT, decarbamoyl gonyautoxin 2 and 3 (dcGTX_2_ and dcGTX_3_) and decarbamoylsaxitoxin (dcSXT) combined (Mix I), gonyautoxin 2 and 3 (GTX_2_ and GTX_3_), gonyautoxin 5 (GTX_5_) and sulfo-carbamoyl saxitoxin 1 and 2 (C_1_ and C_2_) combined (Mix II) were pre-oxidized with hydrogen peroxide and neosaxitoxin (NEO), gonyautoxin 1 and 4 (GTX_1_ and GTX_4_) combined (Mix III) were oxidized with periodate. Standards were obtained from NRC Certified Reference Material Program (Institute for Marine Biosciences, Halifax, Canada). The three mixtures of standards were diluted 10-fold in water (MilliQ), a calibration curve was made with subsequent dilutions in water with five points for each mixture. Concentrations ranging from 6 ng/mL to 257 ng/mL for Mix I, from 8 ng/mL to 354 ng/mL for Mix II and from 6 ng/mL to 290 ng/mL for Mix III. Every calibration solution was made daily for each set of analysis. PSTs were identified by comparison of oxidation products of the standards retention times. PSTs were quantified by direct comparison of peak areas with the calibration curves. Quantified levels for each analog must be converted in STX 2HCl equivalents ng/g, using applied TEFs for each analog [14]. Retention times were: dcGTX_2,3_ (3.75 and 4.05 min), C_1,2_ (5.2 min), dcSTX (7.06 and 7.92 min), GTX_2,3_ (9.17 min), GTX_5_ (10.6 min), SXT (11.8 min) and NEO (8.05 min) (Figure 10). Due to the complexity of the samples and to overcome the matrix effect, non-oxidized samples of each species were injected in the HPLC. Blanks were prepared daily. The limits of detection (LOD) and quantification (LOQ) for the HPLC-FLD are displayed in Table 6.

**Table 6:** Limits of detection (LODs) and quantification (LOQs) of the method for each toxin (expressed in ng/mL).

Toxin	LOD	LOQ
dcGTX_2,3_	2.7	8
C_1,2_	3	11
dcSTX	0.2	6
GTX_2,3_	1.5	9
GTX_5_	0.4	8
SXT	0.4	7
GTX_1,4_	3	9
NEO	1.2	8

#### 3.3.2. Lipophilic Toxins LC-MS/MS Conditions

**Figure 11:**
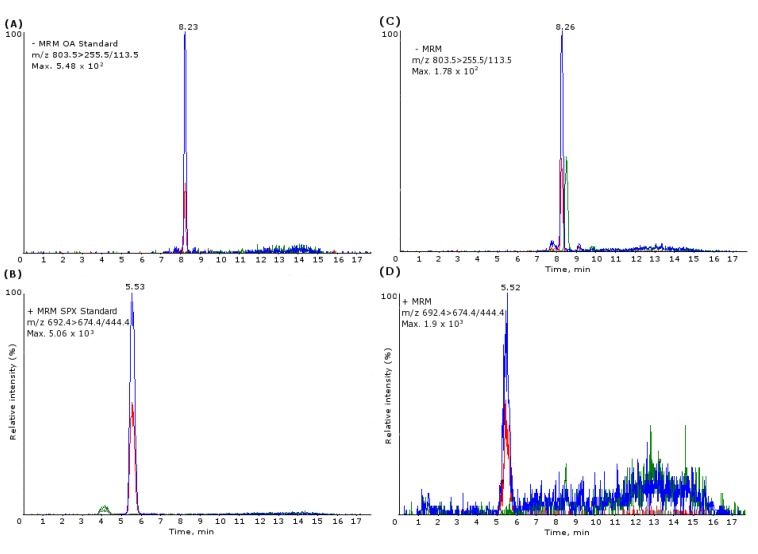
Mass chromatograms of the LC-ESI-CID-MS/MS obtained under multiple reaction monitoring (MRM): (**A**) extracted ion chromatogram (XIC) of OA standard (200 ng/mL), *m/z* 803.5 > 255.5/113.5; (**B**) XIC of 13-desmethyl SPX C standard (200 ng/mL), *m/z* 692.4 > 674.4/444.4; (**C**) XIC of a positive sample for OA in *N. lapillus* (*m/z* 803.5 > 255.5/113.5); (**D**) XIC of a positive sample for 13-desmethyl SPX C in *N. lapillus* (*m/z* 692.4 > 674.4/444.4).

The LC-MS/MS conditions were the same as reported in Otero *et al.* (2011) [70]. Briefly, the analyses were performed with high-performance liquid chromatography (LC) equipment (Shimadzu, Kyoto, Japan) consisting of a binary system of LC-10ADVP pumps, an autoinjector (SIL-10ADVP) with degasser (DGU-14A), refrigerated rack, column oven (CTO-10ACvp) and a system controller (SCL-10Avp). The LC system was coupled to a hybrid quadrupole-linear ion trap mass spectrometer (MS) (2000 QTRAPLC/MS/MS, Applied Biosystems, Carlsbad, CA, USA), equipped with an atmospheric pressure ionization unit (API) and fitted with an electrospray ionization source (ESI). The equipment operated in the conventional mode of low energy of collision induced dissociation (CID) of MS/MS. Nitrogen was produced by a Nitrocraft NC_LC/MS_ generator from Air Liquide (Madrid, Spain). The LC system operated with the ESI interface using the following parameters: curtain gas, 15 psi; collision-activated dissociation gas (CAD), 6 psi; IonSpray voltage, 4000 V; temperature, 450 °C; gas 1, 50 psi; gas 2, 50 psi. Analyst software was used to control the whole process. Toxins separation was performed with a BDS-Hypersil-C8 column (i.d. 2 × 50 mm; 3 μm) and a guard cartridge (i.d. 10 × 2.1 mm) from Thermo (Waltham, MA, USA). Column oven temperature was set at 25 °C. Injection volume of 5 μL. Eluent A of the mobile phase was composed of 50 mM formic acid (Merck, Madrid, Spain) and 2 mM ammonium formate (Sigma Aldrich, Madrid, Spain) in water. Eluent B consisted of acetonitrile (Panreac Quimica, Barcelona, Spain) in water (95:5) with ammonium formate (2 mM) and formic acid (50 mM). The gradient started with 30%–90% of mobile phase (B) for 8 min, then maintained at 90% until min 11, decreasing to 30% over 0.5 min and maintained during 5.5 min until the end of the run. Flow rate was 0.2 mL/min. The mass spectrometer was operated in multiple reaction monitoring (MRM), detecting in positive and negative modes. Two product ions were analyzed per compound, one for quantification and another for confirmation. The transitions employed were: OA and DTX-2 (*m/z* 803.5 > 255.5/113.5), DTX-1 (*m/z* 817.5 > 255.5/113.5), 13,19-didesmethyl SPX C (*m/z* 678.5 > 660.5/430.5), 13-desmethyl SPX C (*m/z* 692.5 > 674.4/444.4). Retention times were: OA (8.2 min), DTX-1 (9.7 min), DTX-2(8.7 min), 13,19-didesmethyl SPX C (4 min) and 13-desmethyl SPX C (5.6 min). For the calibration curve, eight different concentrations of the standard (Laboratorios Cifga, Spain) were injected in duplicate: OA/DTX-1/DTX-2 from 1 ng/mL to 200 ng/mL; SPX from 0.5 ng/mL to 200 ng/mL (Figure 11). All toxins were quantified, using their peak areas to calculate amounts and using the curve obtained from each standard. The LOD and LOQ of the LC-ESI-CID-MS/MS for each toxin were: OA/DTX-1/DTX-2 (4/10 ng/mL) and SPX (0.1/0.5 ng/mL). Due to technical reasons, the LC-MS/MS device was not available, so the analyses for DSP’s proceeded in a UPLC-MS/MS (50 samples of 55).

#### 3.3.3. Lipophilic Toxin UPLC-MS/MS Conditions

**Figure 12:**
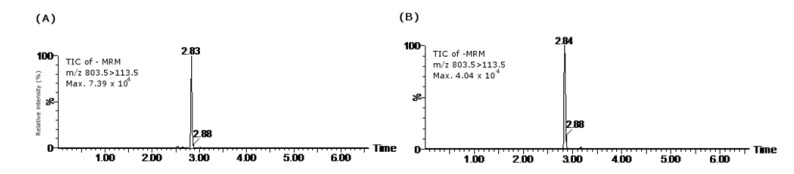
Mass chromatograms of the UPLC-MS/MS obtained under MRM operation: (**A**) total ion chromatogram (TIC) of OA standard 200 ng/mL, *m/z* 803.5 > 255.5/113.5; (**B**) TIC of a positive sample for OA in *M. glacialis*, *m/z* 803.5 > 255.5/113.5.

Samples were analyzed according to Otero *et al.* (2011) [70] with the ultra-high performance liquid chromatography equipment, ACQUITY UPLC system, coupled to a Xevo TQ MS mass spectrometer from Waters (Manchester, UK). The apparatus was equipped with a multimode source ESI/APCI/ESCi, a vacuum system with two air-cooled Edwards Vacuum turbo molecular pumps and one Varian rotary backing pump. The nitrogen generator was a Nitrocraft NCLC/MS from Air Liquide (Madrid, Spain). Chromatographic separation and detection of OA and its derivatives was performed with a Waters Acquity UPLC BEH C_18_ column (100 mm × 2.1, 1.7 μm) with an in-line 0.2 μm Acquity UPLC filter. The column oven was set at 30 °C. Eluent A consisted of water, and eluent B contained acetonitrile (Panreac Quimica, Spain) and water (95:5). Both eluents contained 2 mM ammonium formate (Sigma Aldrich, Spain) and 50 mM formic acid (Merck, Spain). The gradient program used to elute the toxins started with 30% mobile phase B for 3 min, increasing to 90% B over 1.5 min, then kept stable for 1 min and reducing to 30% of B over the next 0.1 min and finally kept for 2 min before the next injection. The flow rate was 0.4 mL/min, and the injection volume was 5 μL. The Xevo TQ MS mass spectrometer operated with the following optimized source-dependent parameters (ESI source): capillary potential 2.5 kV, cone voltage 20 V, desolvation temperature 350 °C, desolvation gas flow 850 L/h N_2_, cone gas flow 50 L/h N_2_, source temperature 120 °C and collision gas flow 20 V. Argon was used as the collision gas at 4.5 × 10^−3^ mbar. The mass spectrometer operated in MRM, detecting in negative mode, analyzing two product ions per compound, one for quantification another for confirmation. The transitions employed were the same used in the LC-MS/MS device; retention times were 2.91 min for OA, 3.08 min for DTX-2 and 3.52 min for DTX-1. For the calibration curve, several dilutions of the standards (Laboratorios Cifga) from 1 ng/mL to 200 ng/mL were set (Figure 12). OA and its derivatives were quantified using their peak areas to calculate amounts and using the curve obtained from each standard. The LOD and LOQ of the method were: 3.7/6.4 ng/mL (OA), 6/10 ng/mL (DTX-1) and 1.6/5.4 ng/mL (DTX-2).

### 3.4. Statistical Analyses

Analyses were performed using R (version 2.14.1) software. In order to test for differences in toxin content for each group of toxins, a general linear mixed model was fitted using toxin concentration as the dependent variable and species and sampling site as additive fixed factors. Seasonal patterns were visually observed in our data distribution (see results), but because the selected dates did not represent the whole possible seasonal effects, the sampling date was included in the model as a random factor. For those fixed factors that showed significant differences, pairwise comparisons between levels were made with the *post hoc* Tukey test. Normality was tested on model residuals with the Shapiro test. When needed, data were normalized by transformation with the Box-cox function. In order to test for differences in toxin content for all toxins together, MANOVA tests were performed using toxin concentration as the dependent variable. Species and sampling site were analyzed as fixed factors in separate models. Hotelling tests were performed for pairwise comparisons between the levels of factors that showed significant differences. Data were tested for normality with a multivariate Shapiro test and normalized with the Box-cox function when needed [71]. Some of the datasets analyzed here contain unbalanced numbers of samples per factor level (see Table 5). This might compromise the accuracy of linear models with complicated factor structures and should be taken into account when interpreting the significance values reported in the tables.

## 4. Conclusions

In this work, we surveyed the Atlantic continental coast of Portugal for unconventional vectors of three groups of biotoxins—PSTs, OA and SPX. Using HPLC-FLD, LC-MS/MS and UPLC-MS/MS techniques, we were able to detect SPX for the first time on the Portuguese coast and also new vectors for this group of biotoxins. We report 16 new vectors for these toxins in the North Atlantic. The values obtained for some species, such as starfish, *M. glacialis*, and the gastropod, *N. lapillus*, suggest that toxin transfer along the food chain probably occurred via mussels. There was no significant evidence of geographical patterns in terms of toxin content among the selected species. However, these differences might be found with a stronger sampling effort. The detection of new vectors, particularly those that are potentially used as food resources, suggests that monitoring of marine toxins should be extended to species other than bivalves in order to limit human health risks. The same could be considered for regulated toxins limits, which are usually calculated for edible bivalves, since our study showed that these toxins are bioaccumulated in upper levels of the food chain. We hope that this work contributes towards the establishment of new legislation, especially for cyclic imines.
